# Sociological analysis of the medical field: using Bourdieu to understand the processes preceding medical doctors’ specialty choice and the influence of perceived status and other forms of symbolic capital on their choices

**DOI:** 10.1007/s10459-018-09872-3

**Published:** 2019-01-17

**Authors:** Caroline Olsson, S. Kalén, S. Ponzer

**Affiliations:** 1 Department of Clinical Science and Education, Södersjukhuset, Karolinska Institutet, Forskningscentrum, Sjukhusbacken 10, 118 46 Stockholm, Sweden; 2 Stockholm’S läns Landsting (Stockholm County Council), Stockholm, Sweden; 3 Orthopaedics Department and the head of Research, Education, Development and Innovation, Södersjukhuset (SÖS), Stockholm, Sweden

**Keywords:** Specialty choice, Symbolic capital, Prestige, Status, Content Analysis

## Abstract

Several studies have demonstrated that medical students and doctors rank specialties differently in terms of perceived status and prestige. At the same time some of the specialties have problems with recruiting and retaining staff. This study aimed to understand what constitutes status and prestige in the medical field and how it influences medical doctors’ choice of specialty. By using a sociological perspective and applying Bourdieu’s theoretical concepts of field, symbolic capital and perceived status, we analysed young doctors’ journeys towards their chosen specialty. We conducted 15 in-depth semi-structured interviews. The data was analysed using content analysis. The findings suggest that medical specialties carry different social status. In the field of power, surgery is seen as the most prestigious of all specialties. However, in the future it might be a less attractive choice when young doctors tend to view their profession less as an identity and more like a job. For specialties perceived as low status, the challenge is to raise popularity by better describing to young doctors the characteristics and advantages of these specialties.

## Introduction

A shortage of physicians in some medical specialties is a global issue, including many Western countries. Challenges with recruiting and retaining specialists seem to be most frequent in primary care (Pfarrwaller et al. 2017), psychiatry (Mahoney et al. 2004) and geriatrics (Curran et al. 2015; Maisonneuve et al. 2014). According to previous research, social prestige and status play a vital part in medical doctors’ selection of a specialty (Creed et al. 2010; Luke 2003). Norredam and Album conducted a literature review in 2007 to examine the relationship between prestige and specialty choice. They concluded that there is a hierarchical status of specialties in the perceptions of medical students and medical doctors, with surgery at the top and psychiatry at the bottom (Norredam and Album 2007). Prestige and status are essential to the French sociologist Pierre Bourdieu’s theoretical framework. According to Bourdieu, agents within a field struggle for different forms of capital (i.e. cultural, economic, social or symbolic) to gain the prestige required to be successful within their field (Brosnan 2010; Bourdieu 2011). The agents—in this case, medical doctors—fight over assets to gain attractive positions in the medical field. The hierarchy of medical specialties can be seen as indicators of social prestige. Therefore, Bourdieu’s concepts can be used to investigate physicians’ specialty choices to understand the meanings of prestige and status within the medical field (Hindhede and Larsen 2018).

Sweden has a relatively egalitarian educational system; indeed, there is no cost for higher education (Börjesson et al. 2016). Specialty choices take place after medical school, and the licence to practise makes it possible to apply for any specialty. Specialty training is undertaken within a framework of employment, and there are almost no differences in salary due to one’s specialty. Accordingly, we were interested in investigating if perceptions of status and prestige affected specialty choices in an egalitarian system like Sweden’s. Therefore, we (Olsson et al. 2018) conducted a quantitative study in which we measured the perceived status of eight specialty groups among Swedish medical doctors (n = 262) using a Likert scale-type question ranging from very high status (1) to very low status (6). The statistical analysis revealed major perceived status distinctions for the eight specialty groups included in that investigation. Surgery was valued as having high status by 69% of respondents. Only 6–7% of respondents considered geriatrics, psychiatry and laboratory specialties to have high status. We also found that high status was associated with one’s choice of specialty. However, the results of that work did not contribute to a deeper understanding of what constitutes status and prestige within the medical field and its influence on specialty choice. Consequently, we decided to continue our investigation with the present study.

Extensive research has targeted medical doctors’ choice of specialty, but most studies have used quantitative methods and a research approach that does not focus on the *process* of how choices are made (Pfarrwaller et al. 2017). One early attempt to problematise the *process of choice* with a theoretical framework of choice itself was conducted in 1997 by (Burack et al. 1997) They concluded, ‘Little attention has been paid to how choosers choose’ (Burack et al. 1997, p. 534). In addition, they found that choice should be considered an ongoing process, both conscious and rational and simultaneously unconscious and hard to assign to a certain moment in time (Burack et al. 1997).

### Bourdieu’s educational sociology

From Bourdieu’s theoretical framework, three entwined concepts will be the lenses in our analysis: field, habitus and different forms of capital. Each concept will be explained subsequently in the sections that follow.

### Field

A *field*, like the medical field (Balmer et al. 2017; Luke 2003), should be understood as a social space. It is the context in which agents act and invest to be successful within a specific area (Carlhed 2007, 2011). Agents within a field fight over assets and positions using various forms of capital (Witman et al. 2011).

### Habitus

People’s experiences become embodied in *habitus*, which can be defined as systems of dispositions that enable individuals to act, think and navigate in the social world. As Collyeret al. (2015) put it: ‘The habitus, for Bourdieu, is an explanatory tool that shows how our actions are always historical, for our individual history shapes our thoughts and actions into “durable dispositions” that guide future behaviour’ (Collyer et al. 2015, pp. 205–206). Habitus is shaped in relation to context (i.e. the fields to which a person belongs). Even though family background and upbringing play an important part in the creation of habitus, it should not be considered static and unchangeable. For instance, the education system plays a particular role in developing habitus (Bourdieu and Passeron 1977, 1979). Balmer et al. (2017) revealed that habitus changes as high in the education system as medical school. Habitus provides a means of understanding agents’ possibilities and limitations within the medical field. Accordingly, ‘Habitus can be used as a *research tool* to form a part of an empirical analysis about the culture and formation of dispositions. Habitus interacts with the medical field and ultimately shapes the dispositions and preferences of junior doctors’ (Luke 2003, p. 55 [Our Italics]) [Emphasis added].

### Forms of capital

Bourdieu used three main forms of capital when analysing the social order of a field: economic capital, social capital (networks, groups) and cultural capital. For this study, to better understand the influence of perceived status and other forms of cultural capital in specialty choices, the Swedish egalitarian educational system is a fitting research context since economic capital can almost be ruled out. In Sweden, the economic factors (i.e. salary, benefits) are quite the same, regardless of specialty.

Most interesting for this study is, however, the role of symbolic capital (Bourdieu 2011). Symbolic capital reveals the contextual nature of cultural capital, meaning that an asset (e.g. taste, manners or cultural knowledge) must be given value within a specific context to be meaningful. In other words, what is recognised as important within a specific field evolves to constitute symbolic capital and indicates prestige or high status for those within that field (Chernilo et al. 2013; McDonald 2014).

The medical profession can be considered ‘closed’; that is obtaining access to the profession requires formal competences and a licence to practise (Lindgren et al. 2011). This fact contributes to the feeling the medical profession is a ‘world of its own’ where investments and power struggles within the profession are what count (Bourdieu 2013). In other words, to regard the medical field as a field of power in a Bourdian sense allows us to investigate which assets are important to physicians (Brosnan 2010).

The aim of this study, then, was to obtain a deeper understanding of processes that precede medical doctors’ choice of specialty and to investigate the influence of perceived status and other forms of symbolic capital on that choice.

## Methods

The research process was undertaken in the qualitative methods tradition. The epistemology (Bunniss and Kelly 2010) behind this study was a mix of interpretivism and critical theory. That means that we consider knowledge as subjective and based on different interpretations, but simultaneously, as something co-constructed between individuals and groups. Critical theory also provides an opportunity to take in varied power relations, which guided our choice of using Bourdieu’s concepts as a theoretical framework. As for the gathering of data, we used in-depth interviews. For the analyses, we took an interpretive approach, performing content analysis (Patton 2015).

### Data collection and participants

A purposeful sample strategy (Patton 2015) was used to gain rich data by variations in terms of participants’ gender and chosen specialty (Table 1). The participants were between 30 and 41 years old; the median age was 33. Inclusion criteria were that participants should be undertaking specialty training at the time of the interview and have been doing so for at least 18 months. Participants were recruited via e-mails sent to the department heads of workplaces providing specialty training for doctors. Department heads forwarded the invitation to doctors in specialty training at the department. The participants contacted the researcher directly, thus obtaining anonymity. The data collection was completed during 2017 in Stockholm County and consisted of 15 semi-structured individual interviews (Lingard and Kennedy 2010) with physicians undergoing their specialist training.

**Table 1:** List of Participants

Specialty	Female	Male
Primary care	1	1
Internal medicine	1	2
Geriatrics	2	1
Psychiatry	2	1
Surgical specialties	2	1
Hospital service specialties	0	1
Total	8	7

An interview guide (Patton 2015) was constructed by the first author and discussed in the research group. After two pilot interviews, select modifications were made (Elo et al. 2014). The interview began with two open questions: ‘Tell me about your specialty choice’ and ‘Describe how you came to the conclusion that you wanted to become an (X) specialist. What was important to you’? The interview then specified questions about status and prestige, networks within the profession, personality and specialties the informant had considered. All interviews were conducted by the first author (CO), audio recorded and later transcribed verbatim. The longest was 100 min and shortest interview was 39 min long. The interview data was 15 h and 40 min in total. The research group estimated that about 12–18 interviews would generate sufficiently rich data to answer the research question and be manageable. The data were collected iteratively, with plenty discussions in the research group based on the short notes the first author wrote directly after each interview. The research group discussed the quality of data, whether changes in the interview guide were needed and the amount of data. After 15 interviews, the research group concluded that this purpose was reached (Bengtsson 2016).

### Ethics committee approval

This research was performed in accordance with the Helsinki Declaration. All participants received an informational letter that clearly stated participation was voluntary, that all data was confidential and that the given consent could be withdrawn at any time, without explanation. Hence, written informed consent was obtained from all participants. The Regional Ethical Review Board in Stockholm concluded that no ethical permission was required according to Swedish law (registration number 2017/699-31/5).

## Analysis

Even though the research question in this study was partly theory-driven, the analysis process was conducted with an inductive approach inspired by Graneheim et al. (2017) and Graneheim and Lundman (2004), meaning that codes were derived from the data and not determined beforehand.

Short notes were written directly after each interview as a tool for remembering the participant and circumstances of the interview, and the interviewer discussed the content with the research group. When all the interviews were completed, the first author read the transcripts, highlighting meaningful data by making notes in the margin. The audio recordings worked as support, and some sections of interviews were listened to while coding the data to capture the underlying meanings of sighs, laughter, hesitations and other non-verbal communication. The process was iterative, going back and forth between the data (interviews) and the coded content. Both the manifest and latent content of interviews was analysed (Graneheim et al. 2017). While a manifest analysis stays close to the text, analysing what the informant actually says, the latent part of the analysis, which involves a greater level of interpretation, makes it possible to capture the underlying meaning the participant infers (Bengtsson 2016). The content was first coded with open coding and then abstracted into sub-categories and main categories. From sub-categories and main categories, themes emerged, capturing the latent, underlying meaning of content. Finally, all interviews were re-read to ensure content was not missed. All the steps in the analysis process were conducted by the first author and discussed within the research group until a consensus was reached, as recommended by Elo et al. (2014). All transcripts were coded in NVivo 11 Pro for Windows.

Moreover, trustworthiness is central when conducting research using qualitative methods (Lincoln 1985). Accordingly, we reported on sampling strategy, data collection and analysis methods in detail (Elo et al. 2014). We also believe that this study was strengthened by the diversity in the group of researchers. All parts of this project were discussed several times within the research group; our different backgrounds contributed to lively debates and a constructive process (Elo et al. 2014). The group consisted of one professor of orthopaedics (SP), one registered nurse with a PhD in medical education research (SK) and the first author, a doctoral student in medical education research with a background in educational sociology (CO). Performing all the interviews was the first author (CO), who had considerable knowledge of the medical context from working in a medical university but is not herself a medical doctor.

## Findings

**Fig. 1:**
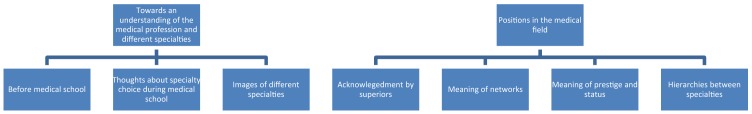
The two themes and main categories

The analysis resulted in two themes: *towards an understanding of the medical profession and different specialties*, based on seven sub-categories and three main categories, and *positions in the medical field,* based on nine sub-categories and four main categories, as shown in Fig. 1.

Together, the themes created a picture of how participants chose their specialty. A useful metaphor when interpreting the results was a journey, even if the findings are not necessarily presented in chronological order. Each theme will be discussed in greater detail henceforth.

### Theme 1: Towards an understanding of the medical profession and different specialties

When the participants describe their journeys to their chosen specialty, they talk about their paths to medicine *per se*. This means that they consider their social background and upbringing important in relation to the medical profession. This theme reveals the participants’ view of the contribution of family values regarding school, the importance of achieving high grades, as well as a parent’s possibility of transferring knowledge about the medical system.

The following quote illuminates the role of upbringing:

‘It was at home—my mother was a teacher and my father an engineer, or chemist, actually, before. And both of them had been to university. And everyone on my Mum’s side of the family and Dad’s, also, really, have been to university, so that was something, we had a culture at home that school was important and you should go on to higher education or similar. And I wanted to, too. But I didn’t know what I wanted to do, and it was first towards the end of school, really, when I finally finished, that I felt I wanted to become a doctor’. (No. 14, geriatrics, man)

The participants discussed how they needed to make educational investments to obtain access to medical school. Many of them worked hard to get good grades and the right formal competences. This quote articulates one of the participants’ efforts:

‘Definitely, of course, you have to choose the right school subjects as you approach senior level, and of course, that I was motivated to study, because I knew the deal: “I’m going to get good grades; otherwise, I’m not going to be able to get in to do the degree I want to do”. So, definitely. If I’d wanted to be something completely different that didn’t require such good grades, then I probably wouldn’t have worked so hard’. (No. 13, primary care, woman)

However, this theme also shows that one’s future specialty choice was not on most people’s agendas before entering medical school. Especially for those participants who did not have close contact with medical doctors (parents/relatives who were doctors), there was a lack of knowledge of how the medical system works:

‘I had no clue about the medical profession, really. I’ve got no doctors amongst my relatives and family or the like. I don’t think I even knew that you chose a speciality, but maybe I had a vague idea that you could focus on one area. But I didn’t have any ideas about what that involved when I started. I don’t think so. I don’t really know when I began to understand how the whole thing is structured. I knew that you could be a paediatrician. I knew that; I’d come across that earlier in life. Yes, as a patient or with my siblings. Yes, so I knew you could be that. But otherwise, I had no idea about all the subdivisions: internal medicine, cardiology and so on. I didn’t really know how it was organised’. (No. 5, internal medicine, woman)

Another part of the journey to become a medical specialist concerned one’s experiences during medical school. During the undergraduate years, the informants discussed their future specialty choice with other students. These discussions were described as having both positive and negative aspects. In one way, it contributed to the upcoming decision, but it also created stress and competition.

‘Yes, we discussed that. There were always some who were pretty certain what they wanted to do and had been from early on during medical school. I wasn’t at all. I wondered about paediatrics, to become a paediatrician or something. But it wasn’t carved in stone in any way. So, yes—we discussed things. I remember being a bit jealous of those who’d made a plan and knew what they wanted. And they could already start to work towards it at medical school. Make sure they got summer jobs as health care assistants on those wards, maybe start some research in that area or something. So in that way, we did discuss things. Definitely’. (No. 5, internal medicine, woman)

When the participants talked about their own specialty, they tended to use words or sentences that were either descriptive, such as ‘It’s a broad specialty’, or words or sentences that express a contrast, like ‘It is not like in surgery, which is a highly competitive speciality’. Frequent dichotomies were used to illustrate these differences when describing specialties, for example ‘To operate vs. not to operate’, ‘Hands-on vs. intellectual work’, ‘To compete vs. not worth the effort’ and ‘On-call duties vs. no on-call duties’. The process of choosing seemed to start with these simple contrasting images, and these images were often given value in relation to surgery. One aspect that divided the participants in their choices was an attraction towards hands-on work or more intellectual parts of medicine. For the participants that chose internal medicine or psychiatry, such analytic parts were considered important. Stated one participant, a trainee in psychiatrics:

‘Understanding context, complexities, this I thought was kind of fun. This is why I liked physiology, and cardiology as well, that there was a kind of a logical coherence that one had to sort of grasp. …///…And that is, to a large extent, the character of psychiatry, that it is kind of unexplored, that it is complex, sort of a unity and so many factors that matter’. (No. 7, psychiatry, man)

For others, such as trainees in surgery and in hospital service specialties, hands-on sorts of work were more attractive:

‘I have to do something with my hands; I have to. We spend limited time seated. We examine quite a lot with our hands, and we actually touch the patients now at the lab. But yes, I have to do something, you know, not just sit and think’. (No. 10, laboratory medicine, man)

The desire to become a surgeon concerned operating per se, whether one found it interesting or even fun to operate. However, it is also connected to the informant’s idea of what is needed to become a (good) surgeon and the will to make the necessary investments.

‘It’s a really fun job. Particularly the actual operating, particularly when you feel you have the time to do what you have to do…//…Yes, the number, the proportion of days that I don’t get away on time at the end of a normal working day are more than those that I do get away. Sometimes, it’s because I’m in the operating theatre and I think I’m learning, so it feels worth it. Sometimes, it’s due to other reasons, and then maybe I don’t think it’s worth it’. (No. 1, orthopaedics, woman)

### Theme 2: Positions in the medical field

Almost all the participants, regardless of specialty, said they socialised both privately and professionally with other doctors. Some participants could see a link between their networks and career opportunities. Relationships of a more private character were often maintained with doctors from other specialties; these started during medical school or one’s residency. Intra-specialty relationships were more professional. Many participants said they regularly attend meetings and other events arranged by their specialty association, the union or networks set up for doctors’ specialty training. It was not possible to draw a sharp line between private and professional relationships.

The subjects discussed their experiences of being acknowledged and of feeling desirable as a future colleague. These experiences started during clinical placements in medical school and continued when applying for training positions as specialists. They said that encouragement contributed to positive feelings for a specialty and, therefore, influenced their choice. This quote illustrates how the role of supervisors can impact one’s choice of specialty:

‘Well, probably, yes, but there were many mentors that you had when you went ’round to different departments who were really like, ‘Seriously, you should start in our specialty’! There was a lot of lobbying, like in paediatrics or gynaecology; ‘We need more men here. You should apply’! or ‘You’re good with children. You should start here in Paediatrics’. That kind of thing. Certain specialties are really persuasive’. (No. 3, internal medicine, man)

In the following quote, the participant stated that a proposal from a clinic made her think differently and, in the end, contributed to her choice of specialty:

‘And then I was actually phoned up from the department where I’d been locuming and asked if I wanted a residency. And it suddenly felt like a really good idea! I liked it there. And in light of my previous experiences, I could see the advantages with Geriatrics. In a way that I couldn’t see when I was newly qualified and really wanted to do something exciting, acute situations and that kind of thing. It sorted of developed; other things started to feel at least as important’. (No. 4, geriatrics, woman)

One participant described an informal recruiting process to surgery that starts in medical school and continues when one applies for temporary work and, eventually, a specialist training position. To be considered for a surgeon’s training position, one must perform at a top level in all parts of the journey.

‘The simple reason is that I ended up here as a resident because I’d worked here previously. It’s hard to get in as a resident if you haven’t worked here. It’s kind of a prerequisite…//… It’s the chicken and the egg problem—how do you get your first temporary job? Is it just chance? Presumably, those students that shine have a better chance. But it can be completely unfair. If somebody happens to have a sick child when they take that course and can’t show themselves to be a budding surgeon, something like that, but might otherwise have been the world’s best surgeon. That’s the thing with chance. Who gets to excel at surgery as a medical student? I think it’s a bit of a shaky foundation to base the surgical profession on’. (No. 6, surgery, man)

This theme also showed that participants perceived status differences between specialties. Surgery was the most prestigious of all specialties, whereas psychiatry and geriatrics are considered to have low status. The following quotes exemplify how status was described in relation to surgery. The first quote comes from a participant who is a surgeon; the other quote is from a subject offered a residency in surgery but who turned it down after some hesitation:

‘One is often reminded of that, that within the profession—although maybe it is actually only the case with other surgeons!—that surgery is a certain status symbol …///… which also comes from the fact that you are responsible…///…, you have a lot of power, and you should be aware of that when you talk to a patient before an operation, because you must give due respect to the fact that, in that position or relationship, the patient is very much in the opposite role, with no power at all. And they’re about to lie unconscious on the operating table, literally putting their life in your hands. And I maybe think that maybe contributes to what I certainly experience as the traditional view that surgeons are something extraordinary and powerful. Certainly, many surgeons consider themselves to be so (laughing). But that doesn’t mean that physicians are as important, but…’ (No. 15, surgery, woman)

‘Naturally, I was a bit insecure, now that I had been given the opportunity to become a surgeon, and I guess it was…..Most of the time, it´s rather difficult to get those employments. It's rather popular everywhere to become a surgeon, so in a way, I was singled out from all other medical interns. Those who had a calling clearly outnumbered those summoned. …///…When I took up internal medicine, it felt pretty good to avoid the jammed queue to the theatre and not have to hang around in the afternoons because the operations are delayed….It felt pretty good just letting go of all that’. (No. 2, internal medicine, man)

Surgery was described as highly competitive, in the sense that one must elbow one’s way into the theatre to become a good surgeon. Some of the participants from other specialties said they would have considered a career as surgeons if the conditions were different regarding the competition, workload and on-call duties.

‘At the surgical and orthopaedic departments where I’ve been, you need to be fairly assertive, even bullish, to somehow get the educational experience you need. You have to make sure you get into theatre, struggle, really, and hinder others in your way to becoming a specialist. And that was not something I had any desire to do. Being somewhere where there was a lot of competition, I wasn’t interested in that. At all’. (No. 5, internal medicine, woman)

The participants training to become specialists in geriatrics, psychiatry and primary care talked about having to defend their choice in front of others and for themselves. The reason was that these specialties were considered to have low status. The following quotes illuminate the feeling of having chosen a low-status specialty:

‘Well, when I chose it, it was rather due to a sort of notion that I would work with something that was either narrower and more organ-specific maybe or something to do with acute illness. I think, in my world, that had higher status. Exactly. Yes. If I was really to ransack myself, then I think on some level, it would have felt better if I’d ended up thinking, “Right, I’m going into emergency medicine’. Yes, probably. If it had worked out in my life and I’d only been able to see advantages with it, then I’d probably have felt more proud of myself than about choosing a residency in geriatrics’. (No. 4, geriatrics, woman)

‘No, psychiatry is not something you choose for status, really. If you present yourself for somebody else, you say you’re a doctor rather than a psychiatrist. Or that you’re a doctor in psychiatry. It’s not really so positive in many people’s eyes’. (No. 7, psychiatry, man)

## Discussion

We have argued in this study that one’s choice of specialty should be perceived as an ongoing process, involving many interrelated aspects that medical doctors considered when they chose their specialty. To fully understand these complex relationships, more information about upbringing and social factors would be useful. Nevertheless, we have had the opportunity to shed light on the meaning of background, simply by asking subjects to describe their journeys towards their chosen specialty. To Bourdieu, family background is essential in terms of reproducing the values, norms and cultural capital that lay the groundwork for the development of habitus (Bourdieu and Passeron 1979). Later, the educational system contributes to the making of habitus (Bourdieu and Passeron 1977). Therefore, a good starting point, when one is trying to understand the process of choosing one’s specialty, is to analyse participants’ descriptions of how they believe their family background and experiences at school mattered for their choice of profession. We captured that in the first theme, *Towards an understanding of the medical profession and different specialties*, where it become evident as participants discussed their early thoughts about the medical profession *per se* and their understanding of medical specialties’ varied characteristics. Clearly, the inherited cultural capital of having parents or other close relationships with medical doctors creates an understanding of the medical field. In Sweden, which is largely egalitarian, the medical profession is still ‘inherited’ from parents to a great extent (Peterson 2006). According to the Swedish national agency for statistics (Statistics Sweden), as many as 23% of medical doctors have a parent who is a doctor, compared with the general Swedish population in the same age group, where the number is only 2–3%. To grow up with parents who are themselves doctors also provides knowledge about the distinctive characteristics of specialties, which was also described by the study’s participants. This kind of knowledge is lacking for those who grew up in an environment without close relationships to medical doctors.

In the second theme*, Positions in the medical field,* it became clear that the recognition of others in the medical field contributes to subjects’ choices. To be trusted and given value within the field, as well as having professional and private networks with other doctors, were described as important. For Bourdieu, networks are a form of social capital, and all members in a network benefit from other members’ success. Bourdieu calls this *magical shareholding* (Bourdieu 1998), and it can contribute to young doctors’ careers. For instance, future work positions can be offered by other members of the network. The study’s participants discussed networks both in terms of social activities and career options. To Bourdieu, it would not be possible to draw a sharp line between these two. There is also a need to recognise the importance of being seen and valued by superiors. For younger doctors, advantageous career options can appear while being someone’s protégé. For senior doctors, that can be a way of transforming cultural capital from oneself to a junior doctor (Bourdieu and Passeron 1979).

The distinct characteristics of medical specialties were also important to participants. Here, they often used dichotomies and contrasts to describe specialties. A major division occurred between more intellectual parts of medicine and more hands-on specialties. Both trainees in psychiatry and hospital service medicine emphasised the intellectual parts of their work. Connected to these statements were comments from both primary care and geriatric specialists who talk about using aspects of their abilities and knowledge was a positive component of their future work. Dehn and Eika (2011), who also refer to Bourdieu when investigating specialty choices in Denmark, found that ‘group’ habitus is produced and reproduced within distinct specialties. According to them, gynaecology and obstetrics were connected to values such as equality, empathy and solidarity. However, vascular surgery stressed teamwork and emphasised visible results, whereas general practise was associated with family values and close relationships. They concluded that there must be some harmony between individual doctors’ habitus and specialties’ group habitus to shape an attractive choice.

Perceived status or prestige is important both as a means of holding a position within the medical field and as an asset that eventually will confer further benefits (Witman et al. 2011). Social status and prestige were a topic that some participants had difficulty discussing; indeed, a few did not even understand why this topic was brought up in the interview. These reactions were expressed by participants who themselves belonged to high-status specialties. In contrast, interviewees in low-status specialties did not seem embarrassed. Some even started to talk about the low status of their specialty before this theme was mentioned in the interview. One of the strongest findings of this study was that surgery stands out from the other specialties to such an extent. It was the one specialty to which all the doctors had a relationship. On the opposite side of the spectrum, interviewees training to become specialists in geriatrics, psychiatry or primary care talk about belonging to low-status specialties and how they often had to defend their specialty choice to family, friends and colleagues. Nonetheless, almost all participants were satisfied with their choices.

Notably, not all doctors want to become surgeons. All the specialties are needed in the health care system. The problem is that, within the power-laden field of medicine, they are not given the same value. In terms of recruiting and retaining staff in the future, we highlight two distinct aspects that must be considered. First, all specialties should be appreciated—or as one participant stated: ‘If you want to recruit more geriatricians, then I actually think it is important to raise its status. That’s what I think. Probably by showing through political decisions that care of the elderly is important. And by increasing awareness of what that specialty actually involves’. (No. 5, geriatrics, woman). Second, in changing times when being a doctor is seen less as an identity and increasingly in terms of a job (Diderichsen et al. 2011), even specialties with high status should consider recruitment strategies to stay attractive to young doctors.

## Methodological discussion

Small-scale studies like this one can never claim generalisability; we want to state this was never our intention. The egalitarian, fairly homogenous Swedish educational system makes references to social status and prestige subtler. It is fair to say these factors are probably even more powerful in educational systems that are more diverse, like the UK or the US.

Our analysis employed theoretical concepts developed by Bourdieu. We used the concepts we found most helpful when analysing specialty choices. However, it should be mentioned that other concepts from Bourdieu and other thinkers would have led to us presenting our findings in another way. The use of a theoretical framework has many benefits. First, it illuminates findings and gives a deeper understanding of a phenomenon or a process. Another benefit is that it can help the reader to determine whether findings are transferable to their context (Rees and Monrouxe 2010).

In Sweden, as elsewhere, there is an unequal distribution of men and women among the specialties, even if the number of women is increasing in traditionally male-oriented specialties (Diderichsen et al. 2013). Importantly, this is not a gender-focused study. However, during the entire research process, we maintained gender awareness. Therefore, we decided to include both men and women in all the researched specialties. During the analytic process, we considered if and how the findings were gendered. However, our analysis did not show major distinctions based on gender. It is important, however, to understand that gendered processes might occur and that we could have spotted these with another theoretical framework.

## Conclusion

This study depicts how the creation of habitus and symbolic capital play a part in medical doctors’ process when picking a specialty. To view the choice of specialty as a process enabled us to investigate diverse levels of decision making, considering both conscious and rational elements as well as unconscious ones. What is regarded as desirable involves multiple dimensions such as a person’s identity, thoughts about who they are and want to be, feelings about acknowledgement and desirability, ideas about workload and what should count as competence, with perceptions of status and prestige. In a field where everyone has high cultural capital, distinctions must be manifested in symbolic capital instead. The various specialties are regarded by participants as having intrinsic differences in status which have consequences for the specialists associated with them. In the end, medical habitus concerns what kind of people doctors consider themselves to be—or want to be.
